# New Insights on Glucosylceramide Synthase in Cancer Drug Resistance and Myelosuppression

**DOI:** 10.4172/2167-0501.1000120

**Published:** 2013-09-24

**Authors:** Vineet Gupta, Yong-Yu Liu

**Affiliations:** Department of Basic Pharmaceutical Sciences, University of Louisiana at Monroe, LA 71209, USA

**Keywords:** Glucosylceramide Synthase, Glycosphingolipids, Cancer Stem Cells, Drug Resistance, Myelosuppression, Doxorubicin

Diverging from eliminating tumors, many anticancer agents can result in drug resistance and myelosuppression or bone marrow suppression in patients during the course of chemotherapy. Drug resistance and myelosuppression are two major impediments to the success of chemotherapy. Recent study of Bhinge et al. demonstrates that glucosylceramide synthase (GCS) can determine the opposite effects of doxorubicin on breast cancer stem cells *versus* bone marrow stem cells *in vivo* [1,2]. These observations disclose new insights on GCS in stem cells that are basis of drug resistance and myelosuppression.

During the course of chemotherapy, a group of cancer cells can acquire drug resistance, which severely affects the efficacy, and even leads the treatment to failure. It is well known that anticancer drugs can induce multidrug resistant cells from various cancer cell lines [3,4]. Emerging evidence suggests that anticancer agents may induce cancer stem cells (CSCs), which possess malignant pluripotency for tumorigenesis and inherent resistance to conventional anticancer drugs and radiotherapy [5–8]. Previous studies showed that CSCs were increased in doxorubicin-selected breast cancer cells and paclitaxel-resistant ovarian cancer cell lines [9–11]. Breast cancer stem cells (BCSCs) were reported significantly increased in tumors that did not respond to doxorubicin chemotherapy (doxorubicin plus docetaxel and doxorubicin plus cyclophosphamide) [12]. Our work demonstratesd that doxorubicin (Dox) induced BCSCs in tumors *in vivo* [1]. In human breast cancer, the CD44^+^/ESA^+^/CD24^−/low^ cells have been tested as BCSCs, since they are able to differentiate into cells with diverse phenotypes, and have tumorous pluripotency to generate mammary tumors and metastases *in vivo* [2,5,13]. We examined the effects of Dox on BCSCs in two different conditions, short- and long-term treatments. Primarily, mice bearing orthotropic mammary tumors were treated with Dox for 6 days. It was found that the numbers of BCSCs (CD44^+^/ESA^+^/CD24^−/low^) cells significantly increased with the increasing doses of Dox (1–5 mg/kg, *i.p*.); at the 2 mg/kg and 5 mg/kg of Dox treatments, BCSCs were increased to 150% and 326%, respectively, as compared to saline group. Further, tumor-bearing mice were treated for 42 days with Dox dose (1 mg/kg *i.p*. once a week) that is close to the dose used for cancer patients. It was also found that the BCSC numbers were significantly increased to 145% in Dox group. These results clearly show that Dox induces BCSCs in tumors. In one-week treatment, Dox may increase the percentage of BCSCs in tumors by killing the differentiated cancer cells. However, it is possibly that long-term Dox treatment induces BCSC proliferation, but this requires further study.

Besides the observation on BCSCs, we also assessed bone marrow stem cells (BMSCs, ABCG2^+^) in these tumor-bearing mice after Dox treatments [1]. Decreased BMSCs, which include mesenchymal stem cells and hematopoietic stem cells, are cause of myelosuppression that not only limits the treatments but also is a risk factor for poor prognosis, as it substantially diminishes the immunity [14,15]. Consistent with previous reports that Dox causes myelosuppression [16,17], we found Dox significantly decreased the numbers of BMSCs of tumor-bearing mice either in 6-days or in 42-days treatments [1]. Altogether, this study showed that Dox has the opposite effects, enriching BCSCs but decreasing BMSCs in the tumor-bearing mice (Figure 1).

Characterization of the molecular mechanisms underlying the opposite effects of anticancer agents on CSCs *versus* normal stem cells is critically important. Interestingly, our studies indicate that GCS determines the opposite effects of Dox on BCSCs and BMSCs [1,2] (Figure 1). We found that GCS protein level and enzyme activity in MCF- 7/Dox breast cancer cells (MCF-7/Dox) were 2 times higher than these in bone marrow cells; Dox treatments (0.5 μM) significantly increased GCS expression in cancer cells, rather than in bone marrow cells [1]. In addition to other genes, GCS was reported overexpressed in Dox-selected BCSCs [2,9]. Conversely, treatments of MBO-asGCS, antisense oligonucleotide that specifically suppressed GCS [18,19], defected the opposite effects of Dox on BCSCs and BMSCs in these tumor-bearing mice [1]. GCS is an enzyme catalyzes ceramide glycosylation that converts ceramide into glucosylceramide. GCS is a cause of cancer cells resistance to anticancer agents and is overexpressed in metastatic breast cancer [20–22]. Many anticancer agents, for example Dox, can induce ceramide-mediated apoptosis in cancer cells and in noncancerous cells [23,24]. However, cellular ceramide generated in response to stress, if it cannot kill cells due to low level or non-apoptotic species, may up-regulate GCS expression thus preventing cells from death and endow these cells resistance [25]. Ceramide glycosylation catalyzed by GCS overexpression can protect cells, like BCSCs, from ceramide-induced apoptosis.

GCS is a limiting-enzyme that catalyzes the first glycosylation reaction for synthesis of glycosphingolipids (GSLs) [20,26]. Among GSLs, ganglio-series and globo-series GSLs are associated with the pluripotency of stem cells [2,27,28]. Following GCS overexpression, our work showed that globo-series GSLs, particularly globotriaosylceramide (Gb3) was significantly higher in induced BCSCs than in non-stem cell subsets, and silencing GCS or Gb3 synthase eliminated the pluripotency of induced BCSCs (iBCSCs) [1,2]. Battula et al. [28] reported that ganglioside GD2 (a ganglio-series GSLs) was a marker to identify BCSCs, and GD3 synthase (produces GD2) was overexpressed in human BCSCs; knockdown of GD3 synthase using siRNA or triptolide abrogated tumor formation and mammosphere formation of BCSCs *in vivo*. GSLs are not uniformly distributed in the plasma membrane and are mainly located in the lipid rafts or glycosphingolipid enriched microdomains (GEM) where they interact with various proteins, thus playing an important role in the signal transduction involved in the epithelial-mesenchymal transition (EMT) [29,30]. Our work shows that GSLs maintain BCSCs through activation of c*Src* and β-catenin signaling. Silencing of GCS and Gb3 synthase, and inhibition of β-catenin recruitment decreased the expression of FGF-2 and Oct- 4, which are essential factors for stem cells, and significantly reduced the cancer pluripotency of iBCSCs [2]. It is still far to understand how ganglio-series and globo-series GSL interact with other molecules in the GEM to regulate cellular signaling pathways. At least, we know GCS and GSLs play crucial roles in regulating CSCs as well as normal stem cells, like bone marrow stem cells. Targeting GCS or other enzymes in GSL synthesis may discover new therapeutic approaches improving cancer treatments.

## Figures and Tables

**Figure 1:**
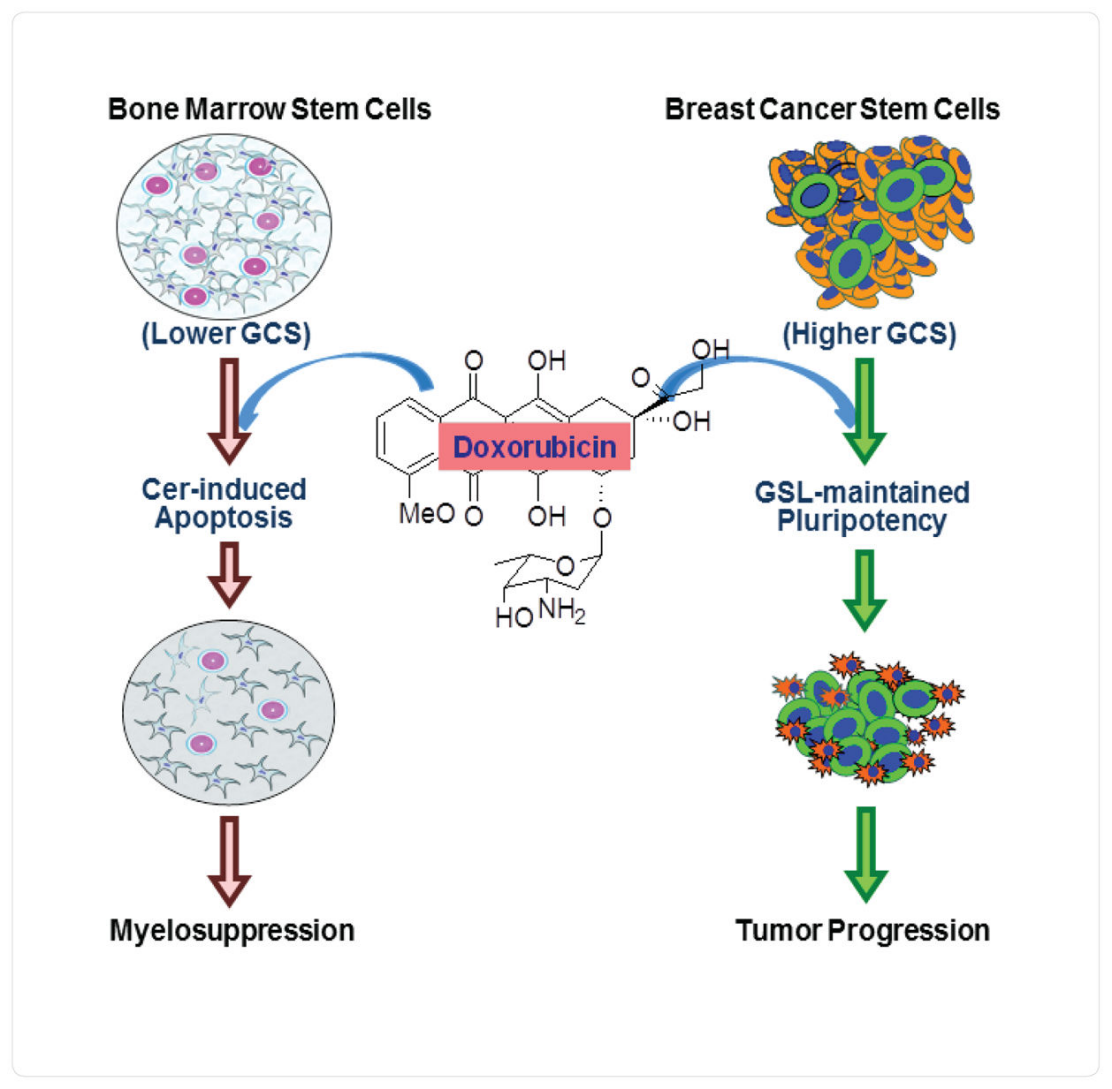
GCS determines the opposite effects of doxorubicin in cancer stem cells versus bone marrow stem cells. Doxorubicin treatments result in cer-induced apoptosis of bone marrow stem cells that have lower levels of GCS, but higher levels of GCS protect cancer stem cells from doxorubicin via GSL-maintained pluripotency. Cer, ceramide; GSL, glycosphingolipid.
